# HIV entry blocked by maraviroc can cause an overestimation of viral load

**DOI:** 10.1186/1758-2652-13-S4-O46

**Published:** 2010-11-08

**Authors:** RD Sloan, BD Kuhl, VG Kramer, DA Donahue, T Bar-Magen, R Tressler, MA Wainberg

**Affiliations:** 1 Lady Davis Institute, McGill AIDS Centre, Montreal, Canada

## Purpose of the study

As a chemokine coreceptor antagonist, maraviroc (MVC) inhibits HIV infection by preventing viral entry into the host cell. As this effect is extracellular, we hypothesized that virus might be returned to plasma upon antagonism of CCR5 by MVC and that this thereby influences measurement of plasma viral load by qRT-PCR, in contrast to other drug classes that act intracellularly.

## Methods

PM-1 and TZM/bl cells were infected with titrations of a CCR5 tropic reference virus in the presence of inhibitory concentrations of MVC (500nM), efavirenz (EFV) (500nm), and raltegravir (RAL) (1µM). Viral inoculation varied between 10^4^-10^7^ copies of viral RNA/ml. Cells were centrifuged and supernatant viral load was measured by qRT-PCR for viral RNA at various times from shortly after infection through 48 hours after infection.

## Summary of results

At the highest inoculum used, the amounts of viral RNA detected in culture supernatants following treatment with MVC were consistently higher by ~ 0.5 log copies HIV-1 RNA/ml than found with untreated cells despite no detectable infection in the presence of MVC as revealed by production of p24 Ag in the case of PM-1 cells and expression of luciferase in the case of TZM/bl cells. At lower levels of inoculum, the results were consistent although less striking, but there was always a significant difference in regard to the use of MVC versus other drugs. In contrast, the results obtained with EFV and RAL resembled those obtained in the absence of drug, despite no detectable infection.

## Conclusions

These results suggest in a tissue culture model that MVC can return virus to plasma, where it may contribute to viral load despite complete inhibition of infection. In contrast, for drugs that act at an intracellular level, cells can absorb residual virus before such virus it is inhibited by the compound. Consequently, such virus will not contribute to viral load. In view of the fact that viral production by infected cells is an ongoing process, these findings imply that the true effectiveness of MVC is currently underestimated when viral load is used as a sole indicator of clinical success. These data may also be relevant for other classes of entry inhibitors.

